# Prolonged dual antiplatelet therapy after drug-eluting stent implantation in patients with diabetes mellitus: A nationwide retrospective cohort study

**DOI:** 10.3389/fcvm.2022.954704

**Published:** 2022-08-11

**Authors:** Seung-Jun Lee, Dong-Woo Choi, Choongki Kim, Yongsung Suh, Sung-Jin Hong, Chul-Min Ahn, Jung-Sun Kim, Byeong-Keuk Kim, Young-Guk Ko, Donghoon Choi, Eun-Cheol Park, Yangsoo Jang, Chung-Mo Nam, Myeong-Ki Hong

**Affiliations:** ^1^Severance Cardiovascular Hospital, Yonsei University College of Medicine, Seoul, South Korea; ^2^Department of Preventive Medicine, Yonsei University College of Medicine, Seoul, South Korea; ^3^Cancer Big Data Center, National Cancer Center, National Cancer Control Institute, Goyang, South Korea; ^4^Ewha Womans University College of Medicine, Seoul Hospital, Seoul, South Korea; ^5^Myongji Hospital, Hanyang University College of Medicine, Goyang, South Korea; ^6^CHA Bundang Medical Center, CHA University College of Medicine, Seongnam, South Korea

**Keywords:** drug-eluting stents, diabetes mellitus, dual antiplatelet therapy, coronary artery disease, treatment outcome

## Abstract

**Background:**

Optimal duration of dual antiplatelet therapy (DAPT) in patients with diabetes mellitus (DM) who have undergone drug-eluting stent (DES) implantation is not clearly established. This study sought to impact of DAPT duration on real-world clinical outcome in patients with or without DM.

**Methods:**

Using a nationwide cohort database, we investigate the association between DAPT duration and clinical outcome between 1 and 3 years after percutaneous coronary intervention (PCI). Primary outcome was all-cause death. Secondary outcomes were cardiovascular death, myocardial infarction, and composite bleeding events. After weighting, 90,100 DES-treated patients were included; 29,544 patients with DM and 60,556 without DM; 31,233 patients with standard DAPT (6–12 months) and 58,867 with prolonged DAPT (12–24 months).

**Results:**

The incidence of all-cause death was significantly lower in patients with prolonged DAPT [8.3% vs. 10.5% in those with standard DAPT, hazard ratio (HR) 0.78, 95% confidence interval (CI) 0.72–0.84] in diabetic patients and non-diabetic patients (4.5% vs. 5.0% in those with standard DAPT, HR 0.89, 95% CI 0.83–0.96). The incidence of composite bleeding events was 5.7% vs. 5.4%, respectively, (HR 1.07, 95% CI 0.96–1.18) in diabetic patients and 5.6% vs. 5.0%, respectively, in non-diabetic patients (HR 1.13, 95% CI 1.05–1.21). There was a significant interaction between the presence of DM and DAPT duration for all-cause death (p for interaction, p_int_ = 0.01) that further favored prolonged DAPT in diabetic patients. However, there was no significant interaction between the presence of DM and DAPT duration for composite bleeding events (p_int_ = 0.38).

**Conclusions:**

This study showed that prolonged rather than standard DAPT might be clinically beneficial in diabetic patients with DES implantation.

**Trial registration:**

ClinicalTrial.gov (NCT04715594).

## Introduction

Diabetes mellitus (DM) is a major risk factor for atherosclerotic cardiovascular disease including coronary artery disease (CAD). Therefore, diabetic patients have a greater prevalence of CAD and account for a substantial proportion of percutaneous coronary intervention (PCI) with drug-eluting stent (DES) in daily clinical practice (1). Even though PCI was performed successfully, the prognosis of diabetic patients showed worse clinical outcomes with higher rates of mortality, cardiovascular events and stent thrombosis during long-term follow-up (2). High platelet reactivity and activation, hypercoagulability (prothrombotic state) and suboptimal response to standard antiplatelet agents might be related to a high rate of adverse cardiovascular events in patients with DM (3). Prolonged dual antiplatelet therapy (DAPT) for more than 1 year was proposed to reduce the occurrence of adverse cardiovascular events in diabetic patients in the past (4, 5). However, use of next-generation DESs has markedly improved clinical outcome after PCI in high-risk patients including those with DM (6). A minimum duration of DAPT is currently advocated in professional guideline documents and adopted worldwide for management of patients receiving DES (7–9). The current guideline suggests that DM should not be the only appraised patient-specific feature when deciding upon the type or duration of DAPT (7). Despite the increased risk of adverse clinical events after PCI in patients with DM compared to those without, the data to support the need for prolonged DAPT (>12 months) are not sufficient in the era of next-generation DES. Therefore, we sought to investigate real-world clinical outcomes according to duration of DAPT in diabetic patients who underwent next-generation DES implantation in a large-volume nationwide cohort that covers the entire population who received first- and next-generation DES implantation for CAD in Korea (CONNECT DES cohort registry).

## Methods

This study was a nationwide retrospective analysis of the national health claims database established by the National Health Insurance Service (NHIS) of Korea, which contains claimed medical costs, drug prescription and adherence, use of medical devices including types of DES, and medical history presented as International Classification of Diseases, Tenth Revision (ICD-10) codes of nearly all inhabitants. Most of the Korean population (97.1%) must subscribe to the NHIS, which is a sole insurer managed by the Korean government. Given that NHIS also covers information for the remaining population (2.9%) categorized as medical aid subjects, this cohort is considered to represent the entire Korean population (10). We were also provided the death certificates and ICD-10 codes from the National Institute of Statistics of Korea. This study was approved by the Institutional Review Board of our institute. Informed consent was waived because personal information was masked after cohort generation according to the strict confidentiality guidelines of the Korean Health Insurance Review and Assessment Service. This study is registered at ClinicalTrial.gov (NCT04715594).

### Study population

**Figure 1:**
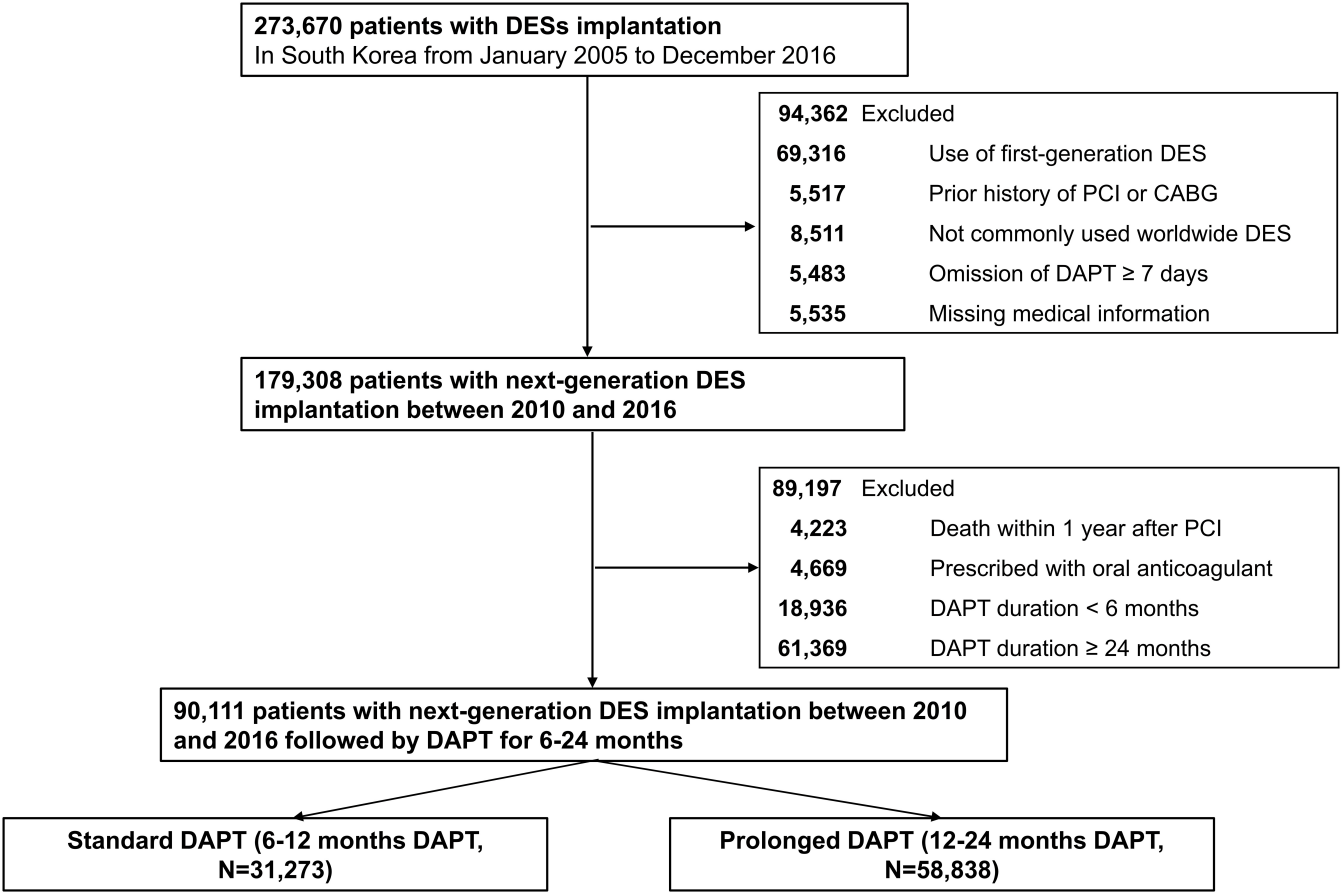
A flowchart of the study population. DES, drug-eluting stent; CABG, coronary artery bypass graft; PCI, percutaneous coronary intervention; DAPT, dual antiplatelet therapy; Standard DAPT, DAPT between 6 and 12 months; Prolonged DAPT, DAPT between 12 and 24 months.

The flow of this study is depicted in Figure 1. From the 52 million inhabitants included in the Korean NHIS database, this study included 273,670 patients (≥20 years old) who were treated with DES between January 2005 and December 2016 in Korea (CONNECT DES cohort registry). The list of included or excluded next-generation DES is presented in Supplementary Table 1. The both types of DESs with biodegradable polymer and durable polymers were included, however, polymer-free DESs were not included in this study. First-generation DES implantation was more frequently performed between 2005 and 2009. Next-generation DESs were more frequently implanted between 2010 and 2016. Following government policy, all information including patients' medical history, drug prescription, and use of medical devices including DES were provided after encryption to protect personal information.

Of the 273,690 patients who underwent DES implantation between 2005 and 2016, 94,362 were excluded for implanted with first-generation DES (*n* = 69,316); previous history of PCI or coronary artery bypass surgery (*n* = 5,517) because clinical events during follow-up cannot be discriminated whether those were caused by a prior PCI (coronary artery bypass surgery) or index PCI; implanted with DES that are not commonly used worldwide (*n* = 8,511); omitted DAPT ≥ 7 days (*n* = 5,483); and missing medical information (*n* = 5,535). Then, 179,308 patients who were treated with next-generation DES remained. To minimize immortal time bias, we excluded those who died within 1 year after PCI from the analyses (*n* = 4,223). Patients who were prescribed with oral anticoagulant (*n* = 4,669), DAPT for <6 months (*n* = 18,936) or DAPT for ≥24 months (*n* = 61,369) were further excluded to minimize selection bias. Consequently, the remaining 90,111 patients who received next-generation DES implantation with DAPT for 6–24 months (standard DAPT, DAPT between 6–12 months, *n* = 31,273; prolonged DAPT, DAPT between 12–24 months, *n* = 58,838, Figure 1) were included in the analyses.

### Study procedures and outcomes

Patients with DM were defined as those who received oral hypoglycemic agents and/or injection of insulin. The duration of DAPT was identified using prescription data with Korean Drug and Anatomical Therapeutic Chemical codes (11). If prescription of aspirin along with any P2Y_12_ inhibitor (clopidogrel, ticagrelor, or prasugrel) has been continued for ≥1 year after index PCI without discontinuation for more than 7 days, we considered the patient to be treated with prolonged DAPT. To minimize immortal time bias, we set the primary follow-up period as 12 to 36 months after index PCI. Patients who experienced ischemic or bleeding events and were alive within 1 year after index PCI were included in the analyses considering the recurrent nature of these clinical events. The history of those clinical events within 1 year after index PCI was adjusted for analyses of primary or secondary outcomes. Primary outcome was all-cause death. Secondary outcomes were composite ischemic events (cardiovascular death, myocardial infarction, or ischemic stroke), composite bleeding events (hemorrhagic stroke, gastrointestinal bleeding, or genitourinary bleeding requiring admission or medical intervention), and each component of an ischemic or bleeding event. Cardiovascular death was ascertained from the National Statistical Office of Korea, which provided death certificates with an accuracy of 92% for the specific cause of death (10, 12). Cardiovascular death was identified by a death certificate with at least one cardiovascular-related diagnosis (acute myocardial infarction, stroke, heart failure, or sudden cardiac death) (13). Myocardial infarction was defined as the simultaneous development of ICD-10 codes corresponding to acute myocardial infarction (11), claims for coronary angiography, admission *via* the emergency department, and more than four rounds of cardiac biomarker testing. A detailed description for each clinical outcome is presented in Supplementary Table 2. We further included baseline comorbidities and drug prescription status before PCI for propensity score calculation, and inverse probability treatment of weighting (IPTW) was used to account for differences in baseline characteristics, medical history, and confounding bias (11, 13). Details regarding covariates included in the propensity score calculation are described in Supplementary Table 3.

### Statistical analysis

Continuous variables are reported as mean and standard deviation, while dichotomous variables are presented as frequency and percentage. Conformity to the normal distribution was assessed for continuous variables using the Kolmogorov-Smirnov test. To minimize the effect of confounding bias, we calculated the IPTW by the propensity score, which was calculated by logistic regression with covariates including age, sex, history of comorbidities and medications, and year of PCI (Supplementary Table 3). We also stabilized the weights by multiplying IPTW by the marginal probability of receiving treatment. The effect size difference between the two groups for all comorbidities and medications was calculated using the standardized mean difference (SMD) and Kernel density plots. SMD values above 0.2 were regarded as a potential imbalance between the two groups. Cumulative incidence curves and the rate of all-cause death, cardiovascular death, myocardial infarction, and composite bleeding events during follow-up were plotted using the Kaplan–Meier method. The adjusted hazard ratio (HR) for each clinical outcome of interest was calculated using a multivariable Cox proportional hazard regression model. The cause-specific hazard model was used to consider death as a competing risk when comparing the incidences of cardiovascular death and other components of secondary outcomes. A two-sided *p*-value of <0.05 was considered significant. Statistical analyses were conducted using SAS version 9.4 (SAS Institute, Cary, NC, USA) and R version 3.6 (The R Foundation, www.R-project.org).

## Results

**Figure 2:**
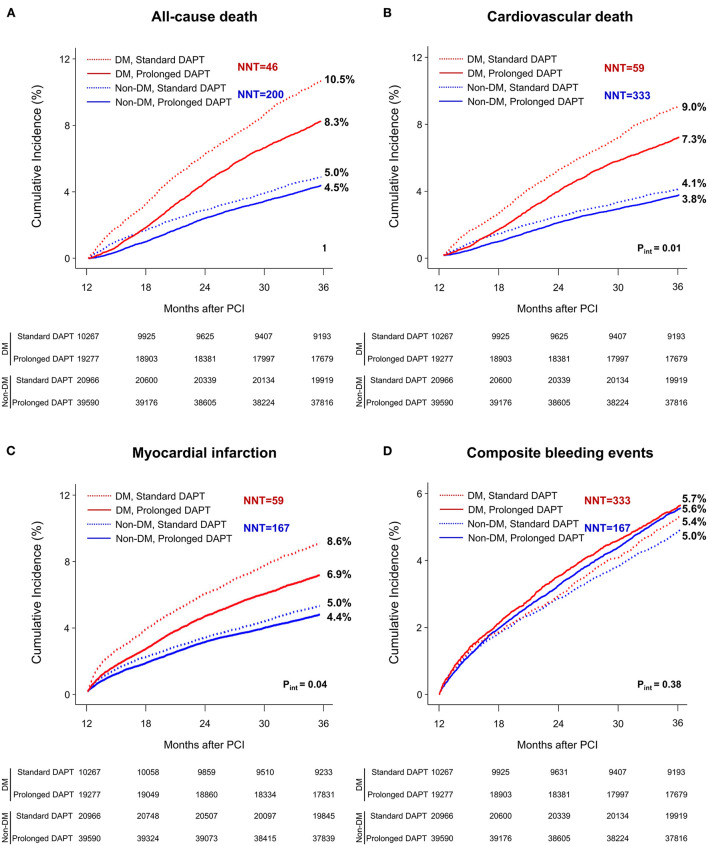
Time-to-event curves for all-cause death, cardiovascular death, myocardial infarction, or composite bleeding events between 1 and 3 years after PCI. The cumulative incidence of **(A)** all-cause, **(B)** cardiovascular mortality, **(C)** myocardial infarction and **(D)** composite bleeding events between 1 and 3 years after PCI. DAPT, dual antiplatelet therapy; DM, diabetes mellitus; PCI, percutaneous coronary intervention. NNT, number need to treat; NNH, number need to harm.

After weighting, 90,100 DES-treated patients included 29,544 patients with DM and 60,556 patients without; 31,233 patients with standard DAPT and 58,867 patients with prolonged DAPT. Baseline demographics and medical history of the whole cohort population before and after stabilized IPTW are presented in Supplementary Table 4. After stabilized IPTW, there was no evidence of inequality in the baseline demographic characteristics and medical history between the two groups including the year of PCI and characteristics of DES (all SMD <0.1, Supplementary Figures 1, 2). Furthermore, baseline characteristics were well balanced among the patients receiving standard and prolonged DAPT with or without DM (all SMD <0.1, Table 1). The incidence and relative hazards of all-cause death, cardiovascular death, myocardial infarction, and composite bleeding events at 2 and 3 years after PCI between the two groups in patients with or without DM are presented in Table 2 and Figure 2.

**Table 1:** Baseline characteristics and medications in patients with and without DM.

**Characteristics**	**Non-DM patients (*****N*** = **60,556)**			**DM patients (*****N*** = **29,544)**		
	**Standard DAPT**	**Prolonged DAPT**	**SMD**	**Standard DAPT**	**Prolonged DAPT**	**SMD**
	**(*N* = 20,966)**	**(*N* = 39,590)**		**(*N* = 10,267)**	**(*N* = 19,277)**	
Age, years	63.7 ± 11.9	63.7 ± 11.8	0.004	66.1 ± 10.9	66.0 ± 10.6	0.013
Women	5,865 (28.0)	10,922 (27.6)	0.009	3,574 (34.8)	6,769 (35.1)	0.006
**Comorbidity**						
Hypertension	12,666 (60.4)	24,007 (60.6)	0.005	7,252 (70.6)	13,517 (70.1)	0.011
Dyslipidemia	8,888 (42.4)	16,906 (42.7)	0.006	4,006 (39.0)	7,492 (38.9)	0.003
Chronic kidney disease with severe renal impairment^a^	675 (3.2)	1,349 (3.4)	0.011	943 (9.2)	1,631 (8.5)	0.026
DM duration ≥ 5 years	-	-	-	6,669 (65.0)	12,736 (66.1)	0.023
Insulin-dependent DM	-	-	-	1,384 (13.5)	2,622 (13,6)	0.004
Heart failure	2,535 (12.1)	4,756 (12.0)	0.002	1,556 (15.2)	2,870 (14.9)	0.007
Chronic liver disease	1,972 (9.4)	3,730 (9.4)	<0.001	1,072 (10.4)	2,058 (10.7)	0.008
Chronic pulmonary disease	1,462 (7.0)	2,715 (6.9)	0.005	732 (7.1)	1,380 (7.2)	0.001
Peripheral arterial occlusive disease	637 (3.0)	1,208 (3.0)	0.001	467 (4.5)	869 (4.5)	0.002
Atrial fibrillation or flutter	541 (2.6)	1,021 (2.6)	<0.001	250 (2.4)	442 (2.3)	0.009
Prior malignancy	871 (4.2)	1,657 (4.2)	0.002	557 (5.4)	1,036 (5.4)	0.002
Prior stroke or TIA	1,516 (7.2)	2,862 (7.2)	<0.001	1,129 (11.0)	2,051 (10.6)	0.012
Prior ICH	115 (0.5)	206 (0.5)	0.004	42 (0.4)	80 (0.4)	0.001
Presentation as AMI	3,869 (18.5)	7,443 (18.8)	0.009	1,697 (16.5)	3,005 (15.6)	0.026
Thyroid disorder	590 (2.8)	1,102 (2.8)	0.002	289 (2.8)	536 (2.8)	0.002
Osteoporosis	1,606 (7.7)	3,007 (7.6)	0.002	803 (7.8)	1,466 (7.6)	0.008
Charlson comorbidity index	1.5 ± 1.3	1.5 ± 1.3	0.002	3.1 ± 1.9	3.1 ± 1.8	0.017
**Medication before PCI**						
Anti-platelet agent	7,680 (36.6)	14,309 (36.1)	0.010	5,367 (52.3)	10,178 (52.8)	0.010
β-Blockers	7,821 (37.3)	14,806 (37.4)	0.002	4,869 (47.4)	9,004 (46.7)	0.014
BP-lowering agents ^b^	4,879 (23.3)	9,098 (23.0)	0.007	2,830 (27.6)	5,393 (28.0)	0.009
RAAS blockade	3,964 (18.9)	7,380 (18.6)	0.007	3,100 (30.2)	5,781 (30.0)	0.004
**Procedural information**						
Number of stents	1.2 ± 0.4	1.2 ± 0.4	0.010	1.2 ± 0.5	1.2 ± 0.5	0.040
**Drug**						
Paclitaxel	3,166 (15.1)	6,116 (15.4)	0.011	1,709 (16.6)	3,118 (16.2)	0.028
Sirolimus	1,807 (8.6)	3,464 (8.7)		943 (9.2)	1,646 (8.5)	
Everolimus	11,861 (56.6)	22,280 (56.3)		5,758 (56.1)	10,924 (56.7)	
Biolimus A9	4,132 (19.7)	7,730 (19.5)		1,857 (18.1)	3,589 (18.6)	
Use of BP-DES	7,298 (34.8)	13,847 (35.0)	0.004	3,570 (34.8)	6,707 (34.8)	<0.001
**Year of PCI**						
2010	1,790 (8.5)	3,399 (8.6)	0.008	834 (8.2)	1,494 (7.8)	0.026
2011	1,517 (7.2)	2,882 (7.3)		746 (7.3)	1,357 (7.0)	
2012	1,366 (6.5)	2,540 (6.4)		668 (6.5)	1,244 (6.5)	
2013	1,749 (8.3)	3,233 (8.2)		794 (7.7)	1,530 (7.9)	
2014	3,271 (15.6)	6,183 (15.6)		1,563 (15.2)	2,882 (14.9)	
2015	4,052 (19.3)	7,637 (19.3)		1,990 (19.4)	3,695 (19.2)	
2016	7,221 (34.4)	13,717 (34.6)		3,668 (35.7)	7,076 (36.7)	

*Values are the mean ± standard deviation or n (%). AMI, acute myocardial infarction; BP-DES, biodegradable polymer drug-eluting stent; BP, blood pressure; DAPT, dual antiplatelet therapy; DM, diabetes mellitus; ICH, intracranial hemorrhage; IPTW, inverse probability of treatment weighting; PCI, percutaneous coronary intervention; RAAS, renin-angiotensin-aldosterone-system; SMD, standardized mean difference; TIA, transient ischemic attack.*

a *Chronic kidney disease with advanced stage requiring intensive medical therapy and financial assistance from health insurance.*

b *Alpha receptor antagonists, calcium-channel blockers or diuretics*.

**Table 2:** Risks of primary and secondary outcomes before and after stabilized IPTW.

		**Non-DM patients**			**DM patients**			***P* for interaction**
		**Standard DAPT**	**Prolonged DAPT**	**HR (95% CI)**	**Standard DAPT**	**Prolonged DAPT**	**HR (95% CI)**	
**Before stabilized IPTW**		***N*** **=** **21,453**	***N*** **=** **39,179**	**Total** ***N*** **=** **60,632**	***N*** **=** **9,820**	***N*** **=** **19,659**	textbfTotal *N* = 29,479	
All-cause death	2 y	605 (2.8)	976 (2.5)	0.88 (0.79–0.97)	602 (6.1)	906 (4.6)	0.74 (0.67–0.82)	0.02
	3 y	1,020 (4.8)	1,747 (4.5)	0.93 (0.86–1.01)	1,012 (10.3)	1,619 (8.2)	0.79 (0.72–0.85)	0.002
Cardiovascular death	2 y	501 (2.3)	838 (2.1)	0.91 (0.81–1.02)	509 (5.2)	809 (4.1)	0.78 (0.70–0.88)	0.06
	3 y	837 (3.9)	1,480 (3.8)	0.96 (0.88–1.05)	862 (8.8)	1,418 (7.2)	0.81 (0.74–0.88)	0.004
Myocardial infarction	2 y	770 (3.6)	1,323 (3.4)	0.94 (0.86–1.02)	669 (6.8)	1,039 (5.3)	0.76 (0.69–0.83)	0.02
	3 y	984 (4.6)	1,734 (4.4)	0.96 (0.89–1.04)	816 (8.3)	1,360 (6.9)	0.81 (0.74–0.88)	0.001
Ischemic stroke	2 y	229 (1.4)	585 (1.5)	1.09 (0.95–1.25)	253 (2.6)	477 (2.4)	0.93 (0.80–1.09)	0.14
	3 y	411 (1.9)	784 (2.0)	1.04 (0.93–1.18)	301 (3.1)	607 (3.1)	0.99 (0.86–1.14)	0.67
Composite ischemic events ^a^	2 y	1,267 (5.9)	2,187 (5.6)	0.94 (0.88–1.01)	1,114 (11.3)	1,833 (9.3)	0.80 (0.74–0.86)	0.001
	3 y	1,728 (8.1)	3,042 (7.8)	0.96 (0.91–1.02)	1,452 (14.8)	2,527 (12.9)	0.84 (0.79–0.90)	0.003
Composite bleeding events ^b^	2 y	667 (3.1)	1,381 (3.5)	1.12 (1.03–1.23)	322 (3.3)	770 (3.9)	1.18 (1.04–1.35)	0.53
	3 y	1,066 (5.0)	2,202 (5.6)	1.12 (1.05–1.22)	527 (5.4)	1,118 (5.7)	1.04 (0.94–1.16)	0.30
Hemorrhagic stroke	2 y	10 (0.05)	24 (0.06)	1.30 (0.63–2.70)	10 (0.1)	19 (0.1)	0.93 (0.43–2.00)	0.54
	3 y	17 (0.08)	42 (0.11)	1.35 (0.77–2.38)	18 (0.18)	29 (0.15)	0.79 (0.44–1.43)	0.21
Gastrointestinal bleeding	2 y	460 (2.1)	953 (2.4)	1.12 (1.01–1.27)	215 (2.2)	553 (2.8)	1.27 (1.09–1.49)	0.21
	3 y	721(3.4)	1,490 (3.8)	1.12 (1.03–1.23)	349 (3.6)	762 (3.9)	1.08 (0.95–1.22)	0.62
Genitourinary bleeding	2 y	208 (1.0)	433 (1.1)	1.14 (0.96–1.35)	103 (1.1)	221 (1.1)	1.07 (0.84–1.37)	0.67
	3 y	360 (1.7)	745 (1.9)	1.13 (1.00–1.29)	181 (1.8)	387 (2.0)	1.07 (0.90–1.28)	0.59
**After stabilized IPTW**		***N*** **=** **20,966**	***N*** **=** **39,590**	**Total** ***N*** **=** **60,556**	***N*** **=** **10,267**	***N*** **=** **19,277**	**Total** ***N*** **=** **29,544**	
All-cause death	2 y	627 (3.0)	985 (2.5)	0.83 (0.75–0.91)	642 (6.3)	896 (4.6)	0.73 (0.66–0.81)	0.08
	3 y	1,047 (5.0)	1,774 (4.5)	0.89 (0.83–0.96)	1,074 (10.5)	1,598 (8.3)	0.78 (0.72–0.84)	0.01
Cardiovascular death	2 y	523 (2.5)	840 (2.1)	0.85 (0.76–0.94)	544 (5.3)	802 (4.2)	0.77 (0.69–0.86)	0.24
	3 y	866 (4.1)	1,497 (3.8)	0.91 (0.84–0.99)	919 (9.0)	1,401 (7.3)	0.79 (0.73–0.86)	0.02
Myocardial infarction	2 y	823 (3.9)	1,323 (3.3)	0.85 (0.77–0.92)	730 (7.1)	1,012 (5.3)	0.72 (0.66–0.80)	0.02
	3 y	1,041 (5.0)	1,735 (4.4)	0.88 (0.81–0.95)	888 (8.6)	1,325 (6.9)	0.78 (0.71–0.85)	0.04
Ischemic stroke	2 y	300 (1.4)	592 (1.5)	1.05 (0.91–1.20)	267 (2.6)	482 (2.5)	0.96 (0.83–1.11)	0.60
	3 y	418 (2.0)	796 (2.0)	1.01 (0.90–1.14)	316 (3.1)	611 (3.2)	1.03 (0.90–1.18)	0.39
Composite ischemic events	2 y	1,316 (6.3)	2,206 (5.6)	0.88 (0.82–0.94)	1,189 (11.6)	1,816 (9.4)	0.79 (0.74–0.85)	0.04
	3 y	1,776 (8.5)	3,074 (7.8)	0.91 (0.86–0.97)	1,545 (15.1)	2,479 (13.0)	0.84 (0.79–0.90)	0.07
Composite bleeding events	2 y	656 (3.1)	1,390 (3.5)	1.12 (1.02–1.23)	348 (3.4)	762 (4.0)	1.17 (1.03–1.32)	0.60
	3 y	1,044 (5.0)	2,218 (5.6)	1.13 (1.05–1.21)	549 (5.4)	1,098 (5.7)	1.07 (0.96–1.18)	0.38
Hemorrhagic stroke	2 y	10 (0.1)	23 (0.1)	1.25 (0.59–2.64)	13 (0.1)	18 (0.1)	0.77 (0.37–1.59)	0.85
	3 y	16 (0.1)	42 (0.1)	1.37 (0.77–2.42)	21 (0.2)	29 (0.1)	0.71 (0.41–1.24)	0.11
Gastrointestinal bleeding	2 y	455 (2.2)	962 (2.4)	1.12 (1.00–1.25)	227 (2.2)	552 (2.9)	1.29 (1.11–1.51)	0.13
	3 y	707 (3.4)	1,501 (3.8)	1.13 (1.03–1.23)	367 (3.6)	751 (3.9)	1.09 (0.96–1.24)	0.71
Genitourinary bleeding	2 y	208 (1.0)	436 (1.1)	1.11 (0.94–1.31)	114 (1.1)	213 (1.1)	0.99 (0.79–1.25)	0.43
	3 y	358 (1.7)	754 (1.9)	1.12 (0.98–1.27)	189 (1.8)	377 (2.0)	1.07 (0.89–1.27)	0.67

*Numbers in parentheses represent the percentage. y indicates year. The hazard ratio (HR) and p value for interaction were calculated by Cox proportional hazard model. CI, confidence interval; DAPT, dual antiplatelet therapy; DM, diabetes mellitus; IPTW, inverse probability of treatment weighting.*

a *Composite of cardiovascular death, myocardial infarction, and ischemic stroke.*

b *Composite of hemorrhagic stroke, gastrointestinal bleeding, and genitourinary bleeding*.

At 3 years after PCI in patients without DM, the incidence of all-cause death, cardiovascular death and myocardial infarction was significantly lower in patients treated with prolonged DAPT [4.5% vs. 5.0% with standard DAPT, HR 0.89, 95% confidence interval (CI) 0.83–0.96; 3.8% vs. 4.1%, HR 0.91, 95% CI 0.84–0.99; 4.4% vs. 5.0%, HR 0.88, 95% CI 0.81–0.95, respectively].

However, the incidence of composite bleeding events was significantly greater in patients treated with prolonged DAPT (5.6% vs. 5.0% with standard DAPT, HR 1.13, 95% CI 1.05–1.21).

At 3 years after PCI in patients with DM, the incidence of all-cause death, cardiovascular death and myocardial infarction was significantly lower in patients treated with prolonged DAPT (8.3% vs. 10.5% with standard DAPT, HR 0.78, 95% CI 0.72–0.84; 7.3% vs. 9.0% HR 0.79, 95% CI 0.73–0.86; 6.9% vs. 8.6%, HR 0.78, 95% CI 0.71–0.85, respectively). There was no statistically significant difference in incidence of composite bleeding events between patients treated with prolonged DAPT and those treated with standard DAPT (5.7% vs. 5.4%, respectively, HR 1.07, 95% CI 0.96–1.18).

The number need to treat for preventing one case of all-cause death was 46 and 200 for patients with and without DM, respectively. The number need to treat or harm for other clinical outcomes among patients with or without DM are presented in Figure 1.

**Figure 3:**
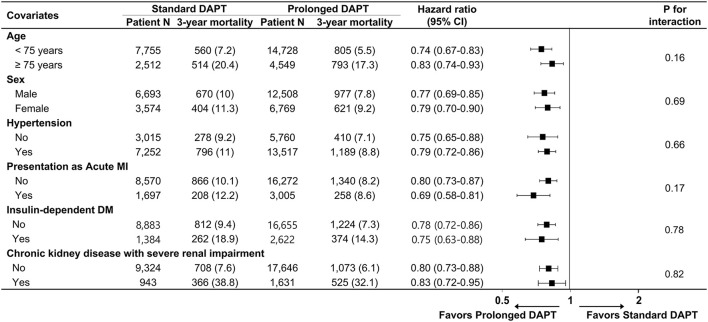
Subgroup analysis for all-cause death in diabetic patients. Numbers and percentages show the number of patients at risk and the all-cause mortality rate between 1 and 3 years after drug-eluting stent implantation. CI, confidence interval; MI, myocardial infarction; DM, diabetes mellitus.

There was a significant interaction between the presence of DM and DAPT duration for all-cause death (p for interaction, p_int_ = 0.01), cardiovascular death (p_int_ = 0.02) and myocardial infarction (p_int_ = 0.04) that favored prolonged DAPT in patients with DM. However, there was no significant interaction between the presence of DM and DAPT duration for composite bleeding events (p_int_ = 0.38). In a subgroup analysis of diabetic patients, there was no significant interaction between the baseline comorbidities and DAPT duration for all-cause death (Figure 3) or cardiovascular death (Supplementary Figure 3).

## Discussion

This nationwide cohort analysis assessed mortality and clinical outcomes of standard vs. prolonged DAPT in diabetic patients with next-generation DES implantation. To the best of our knowledge, the results of our analyses were derived from a cohort that included a largest number of diabetic patients who underwent next-generation DES implantation. This study included whole patients who were concurrently encountered in a catheterization laboratory and were very-high-risk (high bleeding risk, end-stage renal disease, and very elderly patients, etc.) who were usually excluded from other randomized studies. The major findings of our study are as follows: (1) in patients with DM, prolonged DAPT (vs. standard DAPT) was associated with lower all-cause mortality, cardiovascular mortality, and myocardial infarction without an increase in composite bleeding events. (2) In patients without DM, prolonged DAPT (vs. standard DAPT) was associated with a decrease in all-cause mortality and cardiovascular mortality and an increase in composite bleeding events.

Compared to bare-metal stents or first-generation DES, the favorable mechanochemical characteristics of next-generation DES have significantly reduced concern for stent thrombosis (6, 14). In this regard, a growing concern for the risk of bleeding according to prolonged DAPT has emerged as an important issue for long-term management of patients who underwent PCI, and attempts to balance ischemic and bleeding events have led to further shortening of the duration of DAPT (15, 16). A recent meta-analysis with 24 randomized trials reported that extended-term (>12 months) DAPT was associated with a reduced risk of myocardial infarction and a higher risk of major bleeding in comparison with short-term (<6 months) or standard (6–12 months) DAPT (17). There was no significant difference in mortality between the patients with extended-term DAPT and those with short-term or standard DAPT (17). In this regard, the bleeding risk of individual patients, as well as ischemic risk, are taken into consideration for deciding the appropriate length of DAPT (18, 19).

DM is a well-recognized key risk factor for CAD and worse prognosis after PCI (8), which is responsible for systemic atherosclerotic change of the entire vascular structure (20). Therefore, management for DM includes multifactorial life style modification together with intensive medical intervention through glucose lowering agents, lipid-lowering agents, and blood pressure-lowering agents. Indeed, adequate control of DM through sodium-glucose cotransporter-2 inhibitor (21) or glucagon-like peptide-1 receptor agonists (22) are known to reduce the risk of ischemic stroke or cardiovascular death as well as recurrent myocardial infarction, implying that systemic treatment (drugs) rather than local treatment (PCI) is essential for management of diabetic patients with cardiovascular complications. Altered systemic metabolism in patients with DM is associated with hypercoagulability, endothelial dysfunction, and platelet activation, together resulting in a prothrombotic state (23) that possibly requires long-term anti-thrombotic therapy or a more potent P2Y_12_ inhibitor. Furthermore, patients with DM have been reported to have a suboptimal response to aspirin or clopidogrel, probably due to the altered metabolic and pharmacokinetic profile (3, 24).

Despite the theoretical benefit of long-term DAPT in diabetic patients with DES implantation, to date, the clinical benefit of long-term DAPT in the era of next-generation DES has not been clearly demonstrated. At an individual patient-level meta-analysis that compared the clinical outcome between short-term (<6 months) and standard (6–12 months) DAPT in patients with and without DM after DES implantation, standard DAPT resulted in an augmented risk of bleeding without significantly reducing the ischemic events (8). All-cause or cardiovascular mortality within 1 year after PCI were not different among patients treated with short-term or standard DAPT regardless of presence of DM (7). However, in a *post-hoc* analysis of a randomized DAPT trial that investigated the clinical outcome between 12 and 30 months DAPT after PCI, extended DAPT (30 months) was associated with reduced risk of recurrent myocardial infarction in diabetic patients (25). Another *post-hoc* analysis for a randomized DAPT trial identified DM as a significant predictor for future coronary thrombotic events, and DM was incorporated as one of the positive predictors that would benefit from extended DAPT (26). Compared to the present study, part of the study population included in previous randomized studies were patients treated with first-generation DES. The previous randomized studies did not include clinically very-high-risk patients who might be expected to show worse prognosis despite successful DES implantation during long-term follow-up (8, 25, 26). Therefore, the findings of previous studies might have difficulty representing the current situation in an era of next-generation DES. Additionally, in contrast to a previous meta-analysis report from randomized trials that have mostly investigated 1-year clinical outcomes after index PCI in diabetic patients (8), our nationwide cohort analysis investigated the clinical outcome between 1 and 3 years after next-generation DES implantation in diabetic patients. Given that DM is a long-lasting risk factor that continuously hampers prognosis after PCI (27), investigating the clinical impact of DAPT in this period could be of noteworthy importance. Furthermore, the DAPT trial investigated the clinical outcomes between 12 and 30 months DAPT after index PCI excluding the patients who experienced ischemic or bleeding events before 12 months after index PCI (28). Whereas, the present study included patients who were alive and had experienced ischemic or bleeding events within 1 year after PCI and were considered to harbor clinical or procedural risk factors for future hard events (all-cause or cardiovascular death) (29).

In general, prolonged DAPT after PCI is related to significantly increased risk of bleeding compared to short or standard DAPT (30). However, the results of this study demonstrated that the significant reduction of ischemic events by prolonged DAPT in diabetic patients led to favorable outcome including reduced mortality, overwhelming the numerically, but statistically not significant, increased bleeding events. Indeed, there is no obvious correlation between the diabetes and augmented bleeding risk by DAPT after PCI (31). Taken together, the findings indicate that, after DES implantation, prolonged DAPT could be further favored in diabetic patients, as compared with non-diabetic patients to alleviate the risk of recurrent ischemic events and consequent cardiovascular or all-cause mortality.

### Limitations

This study has several limitations. First, observational studies that evaluated the clinical impact of DAPT after PCI are possibly prone to immortal time bias, although we excluded those who died within 1 year after PCI. Second, clinical events that occurred early after PCI or the patient's own characteristics might have influenced the physician's decision for the duration of DAPT. In this regard, there could be persistent residual confounding factors, although we tried to minimize the bias using stabilized IPTW. Third, because the NHIS database does not routinely collect laboratory profiles, the level of glycosylated hemoglobin A1c that represents the severity of DM, was not included as a covariate for the stabilized IPTW model or Cox regression analysis. However, since the Korean health insurance system strictly regulates the use of oral hypoglycemic agent according to the level of glycosylated hemoglobin A1c, it is presumable that the imbalance of DM severity between the two groups would be limited as we defined DM according to the performance of treatment rather than the presence of diagnostic codes. Furthermore, laboratory information regarding platelet reactivity that could give explanation for the suboptimal outcome of diabetic patients after cessation of DAPT was not available. Fourth, contemporary bleeding classification system with prognostic impact [e.g. BARC (Bleeding Academic Research Consortium), TIMI (Thrombolysis in Myocardial Infarction), GUSTO (Global Use of Strategies to Open Occluded Arteries), etc] could not be applied due to limited information. Finally, the occurrence of stent thrombosis was also could not be investigated due to lack of angiographic information. Taken together, the results from this observational study should not be used to establish a causal relationship, until our findings are recapitulated by well-conducted randomized studies.

## Conclusions

In this nationwide cohort of patients treated with new-generation DES in Korea, prolonged DAPT rather than standard DAPT might be clinically beneficial in diabetic patients with DES implantation.

## Data availability statement

The data analyzed in this study is subject to the following licenses/restrictions: The datasets generated for the analyses are not publicly available because of strict government restrictions. Requests to access these datasets should be directed to mkhong61@yuhs.ac.

## Ethics statement

The studies involving human participants were reviewed and approved by Yonsei University Health System. The Ethics Committee waived the requirement of written informed consent for participation.

## Author contributions

S-JL, D-WC, C-MN, and M-KH contributed to the conception and design, verified the data, and conducted all analyses. S-JL and M-KH wrote the study protocol. D-WC and C-MN performed the programming to extract the data from the NHIS database. M-KH and C-MN had full access to all the data in the study and take responsibility for the integrity of the data and the accuracy of the data analysis. All authors have provided a critical review of the manuscript, read, and approved the final publication.

## Funding

This work was supported by the Cardiovascular Research Center, Seoul, Korea. The funder had no role in the design and conduct of the study, data collection, management, statistical analysis, and interpretation of the data, preparation, review, or approval of the manuscript, and decision to submit the manuscript for publication.

## Conflict of interest

The authors declare that the research was conducted in the absence of any commercial or financial relationships that could be construed as a potential conflict of interest.

## Publisher's note

All claims expressed in this article are solely those of the authors and do not necessarily represent those of their affiliated organizations, or those of the publisher, the editors and the reviewers. Any product that may be evaluated in this article, or claim that may be made by its manufacturer, is not guaranteed or endorsed by the publisher.
