# Supplementary material for: Prolonged dual antiplatelet therapy after drug-eluting stent implantation in patients with diabetes mellitus: A nationwide retrospective cohort study
Source: Front Cardiovasc Med. 2022 Aug 11;9:954704. doi: 10.3389/fcvm.2022.954704 (PMC9403781; doi:10.3389/fcvm.2022.954704)
Supplement: Supplementary file 1 [file Data_Sheet_1.docx]

**SUPPLEMENTAL MATERIAL**

**Prolonged dual antiplatelet therapy after drug-eluting stent implantation in patients with diabetes mellitus: a nationwide retrospective cohort study**

Seung-Jun Lee, Dong-Woo Choi, Choongki Kim, Yongsung Suh, Sung-Jin Hong, Chul-Min Ahn, Jung-Sun Kim, Byeong-Keuk Kim, Young-Guk Ko, Donghoon Choi, Eun-Cheol Park, Yangsoo Jang, Chung-Mo Nam, Myeong-Ki Hong

| **Contents** | **Page** |
| --- | --- |
| **Supplemental Table 1.** List of next-generation DES included or excluded from the study | 2 |
| **Supplemental Table 2.** Individual outcome definitions | 3 |
| **Supplemental Table 3.** Covariates include in stabilized inverse probability of  treatment weighting | 4 |
| **Supplemental Table 4.** Baseline characteristics and medications in all patients | 5 |
|  |  |
| **Supplemental Figure 1.** Standardized difference before and after stabilized inverse probability of treatment weighting | 7 |
| **Supplemental Figure 2.** Distribution of stabilized inverse probability of treatment weightings | 8 |
| **Supplemental Figure 3.** Subgroup analysis for cardiovascular death in diabetic patients | 9 |

**Supplemental Table 1.** List of next-generation DES included or excluded from the study

| **Included** | **Excluded** |
| --- | --- |
| **Biodegradable polymer DES** | |
| Ultimaster | DESyne |
| Synergy | Genoss |
| Orsiro | Biomine |
| Nobori |  |
| Biomatrix |  |
| **Durable polymer DES** | |
| Xience Prime | Endeavor Sprint |
| Xience Xpedition | Endeavor ABT-578 eluting stent |
| Resolute Onyx |  |
| Resolute Integrity |  |
| Promus Element |  |
| Promus Element Plus |  |
| Promus Premier |  |

DES, drug-eluting stents.

**Supplemental Table 2.** Individual outcome definitions

| **Outcomes** | **Logical description and ICD-10 codes** |
| --- | --- |
| Cardiovascular mortality | - Cardiac death confirmed by death certificate - Death with ICD-10 codes corresponding to coronary artery disease, acute MI, heart failure or stroke within 1 month |
| Myocardial infarction (MI) | - ICD-10 codes corresponding to acute MI - Performance of coronary angiography within 7 days - Admission via emergency department - Performance of cardiac enzyme check for more than 4 times |
| Ischemic Stroke | Admission for more than 3 days with the following ICD-10 codes : I63, I64 |
| Hemorrhagic stroke | Admission for more than 3 days with the following ICD-10 codes : I60, I61, I62 |
| Gastrointestinal bleeding | Admission for more than 3 days with the following ICD-10 codes : K250, K252, K254, K256, K260, K262, K264, K266, K270, K272, K274, K276, K280, K282, K284, K286, K290, K226 |
| Genitourinary bleeding | Admission for more than 3 days with the following ICD-10 codes : N02, R31 |

**Supplemental Table 3.** Covariates include in stabilized inverse probability of treatment weighting

|  | **Variable list** |
| --- | --- |
| **Comorbidities** | Age, gender, diabetes mellitus, hypertension, dyslipidemia, history of heart failure, presentation as acute myocardial infarction, prior history of stroke or cerebrovascular accident, prior history of intracranial hemorrhage, atrial fibrillation or flutter, prior history of malignancy, hyperthyroidism, hypothyroidism, osteoporosis, chronic liver disease, chronic pulmonary disease, chronic kidney disease with severe renal impairment, year of percutaneous coronary intervention |
| **Medications** | Warfarin sodium, Edoxaban, Rivaroxaban, Apixaban, Dabigatran, Aspirin, Clopidogrel, Prasugrel, Ticagrelor, Atorvastatin, Rosuvastatin, Simvastatin, Pravastatin, Fluvastatin, Pitavastatin, Lovastatin, Tripamol, Hydrochlorothiazide, Chlorthalidon, Indapamide, Metorazone, Furosemide, Torasemide, Amlodipine, Barnidipine, Cilnidipine, Felodipine, Lacidipine, Lercanidipine, Manidipine, Nicardipine, Nifedipine, Nisoldipine, Nitrendipine, Diltiazem, Verapamil, Bunazosin, Doxazosin, Prazosin, Terazosin, Atenolol, Betaxolol, Bevantolol, Bisoprolol, Celiprolol, Metoprolol, Nadolol, Propranolol, Carteolol, Arotinolol, Carvedilol, Labetalol, Sulfonylurea, Metformin, Alpha-glucosidase inhibitors, Thiazolidinedione, DPP-Ⅳ inhibitors, SGLT-2 inhibitors, insulin, Spironolactone, Alacepril, Benazepril, Captopril, Cilazapril, Delapril, Enalapril, Fosinopril, Imidapril, Lisinopril, Moexipril, Perindopril, Quinapril, Ramipril, Temocapril, Candesartan, Eprosartan, Irbesartan, Losartan, Telmisartan, Valsartan, Fimasartan, Azilsartan |

**Supplemental Table 4. Baseline characteristics and medications in all patients**

| **Characteristics** | | **Before stabilized IPTW (N=90,111)** | | | **After stabilized IPTW (N=90,100)** | | |
| --- | --- | --- | --- | --- | --- | --- | --- |
|  | **Standard DAPT**  **(N=31,273)** | | **Prolonged DAPT**  **(N=58,838)** | **SMD** | **Standard DAPT**  **(N=31,233)** | **Prolonged DAPT**  **(N=58,867)** | **SMD** |
| Age, years | 64.1±11.7 | | 64.5±11.5 | 0.034 | 64.5±11.7 | 64.4±11.5 | 0.007 |
| Women | 9,136 (29.2) | | 17,897 (30.4) | 0.026 | 9,438 (30.2) | 17,690 (30.1) | 0.004 |
| **Comorbidity** | | | | | | | |
| Hypertension | 19,432 (62.1) | | 37,998 (64.6) | 0.051 | 19,918 (63.8) | 37,523 (63.7) | 0.001 |
| Dyslipidemia | 12,701 (40.6) | | 24,692 (42.0) | 0.027 | 12,895 (41.3) | 24,399 (41.4) | 0.003 |
| Chronic kidney disease with  severe renal impairment * | 1,717 (5.5) | | 2,819 (4.8) | 0.032 | 1,618 (5.2) | 2,980 (5.1) | 0.005 |
| DM duration |  | |  |  |  |  |  |
| No DM | 21,453 (68.6) | | 39,179 (66.6) | 0.049 | 20,966 (67.1) | 39,590 (67.3) | 0.014 |
| < 5 years | 3,509 (11.2) | | 6,639 (11.3) |  | 3,598 (11.5) | 6,541 (11.1) |  |
| ≥ 5 years | 6,311 (20.2) | | 13,020 (22.1) |  | 6,669 (21.4) | 12,736 (21.6) |  |
| Insulin-dependent DM | 1,232 (3.9) | | 2,764 (4.7) | 0.037 | 1,384 (4.4) | 2,622 (4.5) | 0.001 |
| Heart failure | 3,931 (12.6) | | 7,716 (13.1) | 0.016 | 4,091 (13.1) | 7,627 (13.0) | 0.004 |
| Chronic liver disease | 3,011 (9.6) | | 5,866 (10.0) | 0.011 | 3,044 (9.7) | 5,787 (9.8) | 0.003 |
| Chronic pulmonary disease | 2,120 (6.8) | | 4,126 (7.0) | 0.009 | 2,194 (7.0) | 4,095 (7.0) | 0.003 |
| Peripheral arterial occlusive disease | 1,051 (3.4) | | 2,135 (3.6) | 0.015 | 1,103 (3.5) | 2,077 (3.5) | <0.001 |
| Atrial fibrillation or flutter | 841 (2.7) | | 1,386 (2.4) | 0.021 | 791 (2.5) | 1,463 (2.5) | 0.003 |
| Prior malignancy | 1,485 (4.7) | | 2,638 (4.5) | 0.013 | 1,428 (4.6) | 2,693 (4.6) | <0.001 |
| Prior stroke or TIA | 2,598 (8.3) | | 4,908 (8.3) | 0.001 | 2,645 (8.5) | 4,913 (8.3) | 0.004 |
| Prior ICH | 122 (0.4) | | 312 (0.5) | 0.021 | 157 (0.5) | 286 (0.5) | 0.003 |
| Presentation as AMI | 5,201 (16.6) | | 10,759 (18.3) | 0.044 | 5,566 (17.8) | 10,447 (17.7) | 0.002 |
| Osteoporosis | 2,341 (7.5) | | 4,487 (7.6) | 0.005 | 2,409 (7.7) | 4,473 (7.6) | 0.004 |
| Thyroid disorder | 823 (2.6) | | 1,679 (2.9) | 0.014 | 879 (2.8) | 1,638 (2.8) | 0.002 |
| Charlson comorbidity index | 2.0±1.7 | | 2.0±1.7 | 0.021 | 2.0±1.7 | 2.0±1.7 | 0.006 |
| **Medication before PCI** | | | | | | | |
| Anti-platelet agent | 12,604 (40.3) | | 24,824 (42.2) | 0.038 | 13,047 (41.8) | 24,487 (41.6) | 0.004 |
| β-Blockers | 12,281 (39.3) | | 24,124 (41.0) | 0.035 | 12,690 (40.6) | 23,810 (40.4) | 0.004 |
| BP-lowering agents ^†^ | 7,686 (24.6) | | 14,467 (24.6) | <0.001 | 7,708 (24.7) | 14,491 (24.6) | 0.001 |
| RAAS blockade | 6,837 (21.9) | | 13,277 (22.6) | 0.017 | 7,063 (22.6) | 13,161 (22.4) | 0.006 |
| **Procedural information** |  | |  |  |  |  |  |
| Number of stents | 1.2±0.4 | | 1.2±0.5 | 0.163 | 1.2±0.4 | 1.2±0.4 | 0.010 |
| Use of BP-DES | 11,088 (35.5) | | 20,374 (34.6) | 0.017 | 10,868 (34.8) | 20,553 (34.9) | 0.003 |
| **Year of PCI** | | | | | | | |
| 2010 | 2,875 (9.2) | | 4,578 (7.8) | 0.207 | 2,629 (8.4) | 4,893 (8.3) | 0.010 |
| 2011 | 2,409 (7.7) | | 4,046 (6.9) |  | 2,263 (7.2) | 4,239 (7.2) |  |
| 2012 | 2,011 (6.4) | | 3,734 (6.3) |  | 2,034 (6.5) | 3,783 (6.4) |  |
| 2013 | 2,749 (8.8) | | 4,511 (7.7) |  | 2,544 (8.1) | 4,762 (8.1) |  |
| 2014 | 5,555 (17.8) | | 8,297 (14.1) |  | 4,834 (15.5) | 9,064 (15.4) |  |
| 2015 | 6,493 (20.8) | | 10,839 (18.4) |  | 6,041 (19.3) | 11,332 (19.2) |  |
| 2016 | 9,181 (29.4) | | 22,833 (38.8) |  | 10,889 (34.9) | 20,794 (35.3) |  |

Values are the mean ± standard deviation or n (%). Abbreviations: AMI, acute myocardial infarction; BP-DES, biodegradable polymer drug-eluting stent; BP, blood pressure; DAPT, dual antiplatelet therapy; DM, diabetes mellitus; ICH, intracranial hemorrhage; IPTW, inverse probability of treatment weighting; PCI, percutaneous coronary intervention; RAAS, renin-angiotensin-aldosterone-system; SMD, standardized mean difference; TIA, transient ischemic attack.

* Chronic kidney disease with advanced stage requiring intensive medical therapy and financial assistance from health insurance.

^†^ Alpha receptor antagonists, calcium-channel blockers or diuretics.

**Supplemental Figure 1.** Standardized difference before and after stabilized inverse probability of treatment weighting. The standardized mean difference for each covariate before (green) and after (red) stabilized inverse probability of treatment weighting are presented. Standardized mean difference lesser than 0.10 was considered to be balanced.


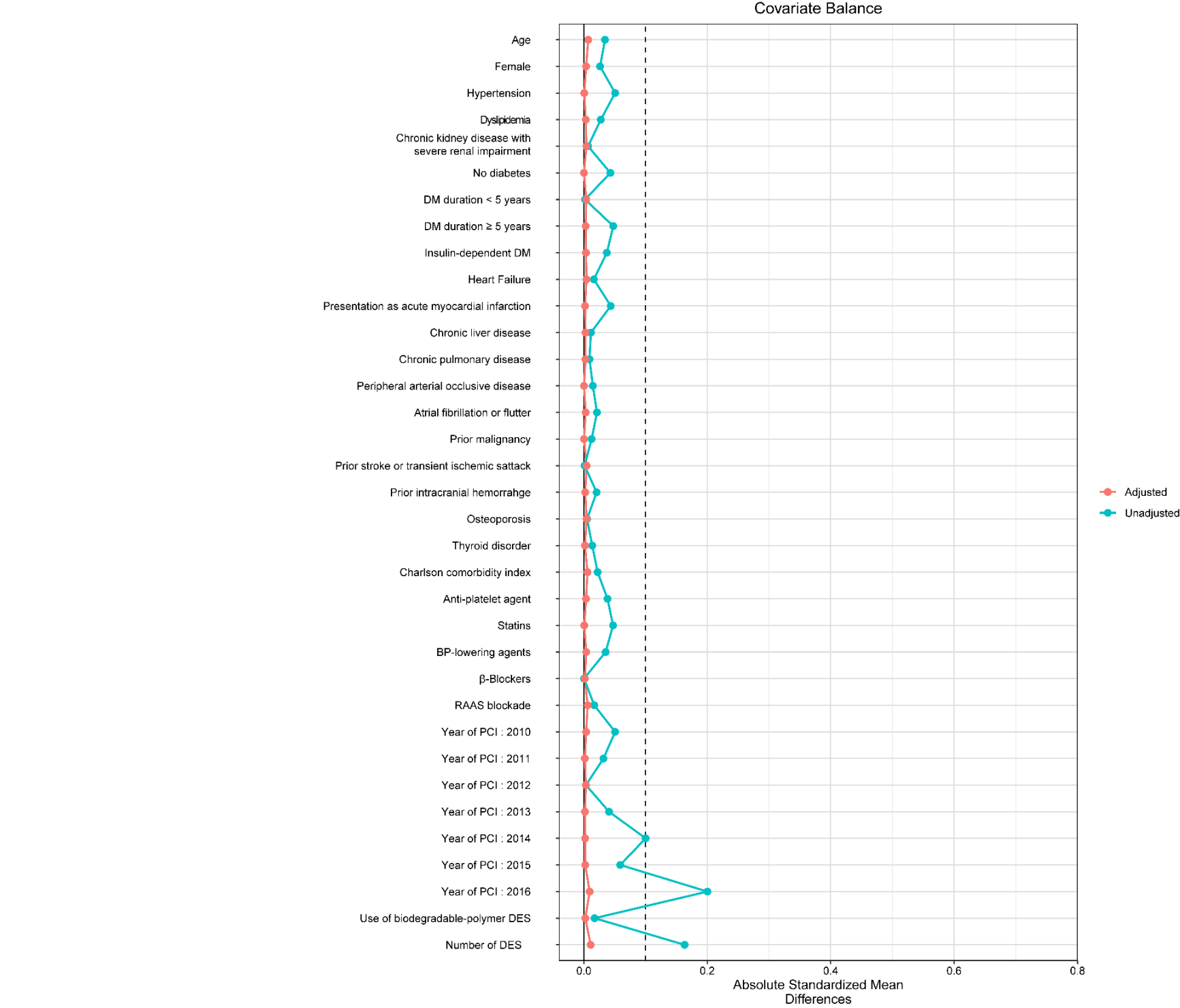


DM, diabetes mellitus; RAAS, renin–angiotensin–aldosterone system; PCI, percutaneous coronary intervention; DES, drug-eluting stent

**Supplemental Figure 2.** Distribution of stabilized inverse probability of treatment weighting

Density plot depicted as red color and blue color indicates standard and prolonged DAPT, respectively.

**
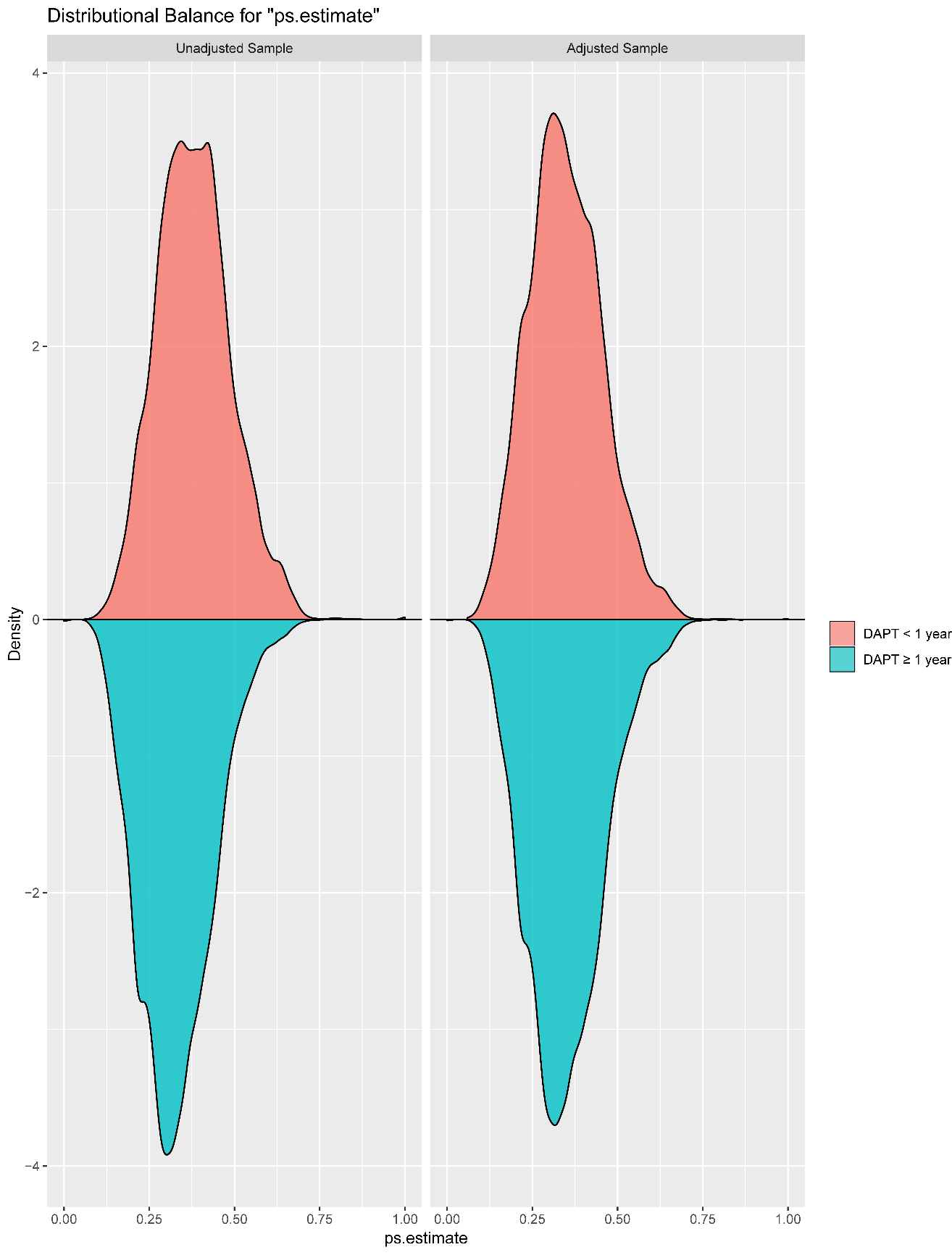
**DAPT, dual antiplatelet therapy

Prolonged DAPT

Standard DAPT

**Supplemental Figure 3.** **Subgroup analysis for cardiovascular death in diabetic patients**


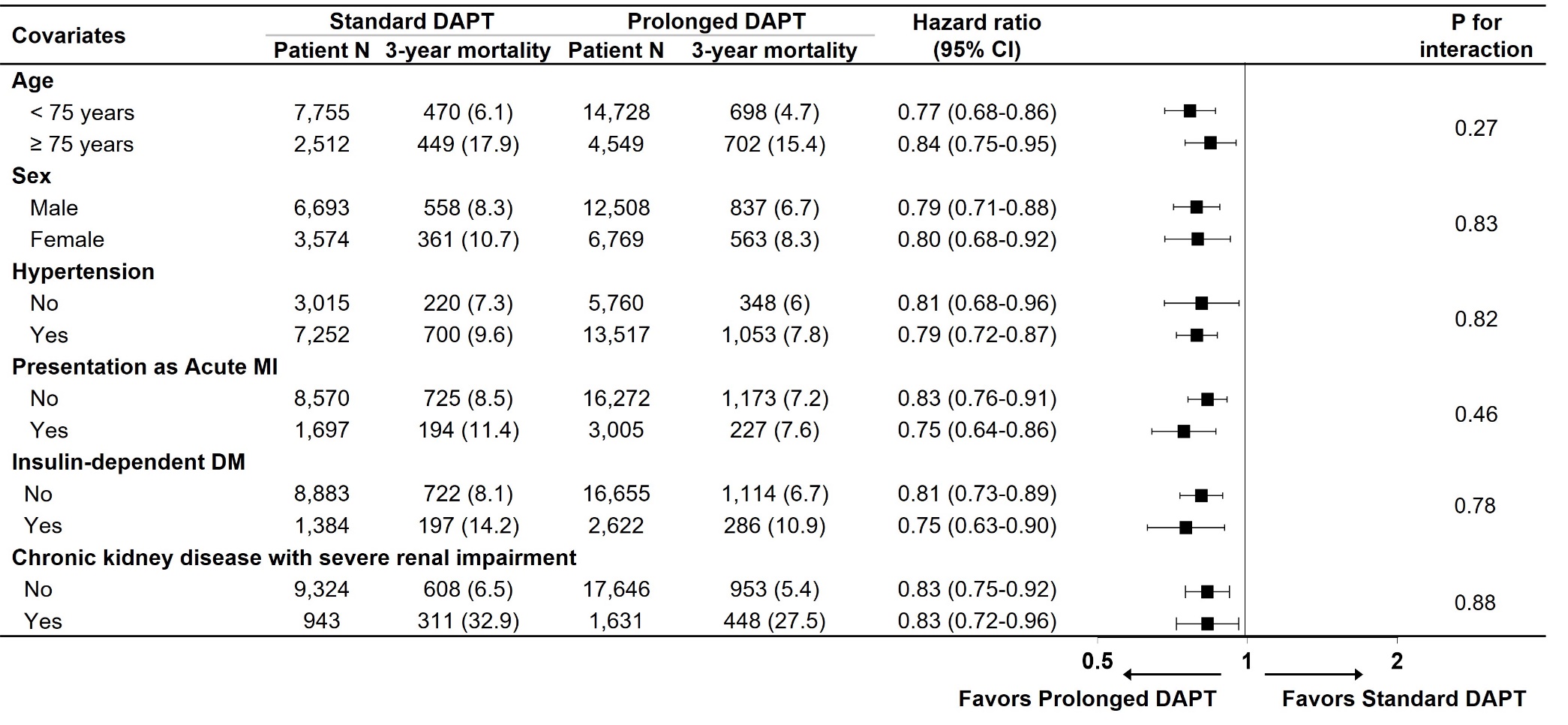


Numbers and percentages show the number of patients at risk and the cardiovascular mortality rate between 1 and 3 years after drug-eluting stent implantation. CI, confidence interval; MI, myocardial infarction; DM, diabetes mellitus.
